# Alleviation of salt-induced exacerbation of cardiac, renal, and visceral fat pathology in rats with metabolic syndrome by surgical removal of subcutaneous fat

**DOI:** 10.1038/s41387-020-00132-1

**Published:** 2020-08-10

**Authors:** Kiyoshi Aoyama, Yuki Komatsu, Mamoru Yoneda, Shiho Nakano, Sao Ashikawa, Yumeno Kawai, Xixi Cui, Katsuhide Ikeda, Kohzo Nagata

**Affiliations:** Pathophysiology Sciences, Department of Integrated Health Sciences, Nagoya University Graduate School of Medicine, Nagoya, Japan

**Keywords:** Cardiovascular diseases, Cardiovascular diseases

## Abstract

**Objectives:**

Evidence suggests that visceral adipose tissue (VAT) and subcutaneous adipose tissue (SAT) should be considered as distinct types of white fat. Although VAT plays a key role in metabolic syndrome (MetS), the role of subcutaneous adipose tissue (SAT) has been unclear. DahlS.Z-*Lepr*^fa^/*Lepr*^fa^ (DS/obese) rats, an animal model of MetS, develop adipocyte hypertrophy and inflammation to similar extents in SAT and VAT. We have now investigated the effects of salt loading and SAT removal on cardiac, renal, and VAT pathology in DS/obese rats.

**Methods:**

DS/obese rats were subjected to surgical removal of inguinal SAT or sham surgery at 8 weeks of age. They were provided with a 0.3% NaCl solution as drinking water or water alone for 4 weeks from 9 weeks of age.

**Results:**

Salt loading exacerbated hypertension, insulin resistance, as well as left ventricular (LV) hypertrophy, inflammation, fibrosis, and diastolic dysfunction in DS/obese rats. It also reduced both SAT and VAT mass but aggravated inflammation only in VAT. Although SAT removal did not affect LV hypertrophy in salt-loaded DS/obese rats, it attenuated hypertension, insulin resistance, and LV injury as well as restored fat mass and alleviated inflammation and the downregulation of adiponectin gene expression in VAT. In addition, whereas salt loading worsened renal injury as well as upregulated the expression of renin–angiotensin-aldosterone system-related genes in the kidney, these effects were suppressed by removal of SAT.

**Conclusions:**

SAT removal attenuated salt-induced exacerbation of MetS and LV and renal pathology in DS/obese rats. These beneficial effects of SAT removal are likely attributable, at least in part, to inhibition of both VAT and systemic inflammation.

## Introduction

Metabolic syndrome (MetS) is characterized by chronic low-grade inflammation and insulin resistance associated with visceral obesity. Cardiovascular damage (left ventricular (LV) hypertrophy, diastolic, and systolic dysfunction) is more frequent in hypertensive individuals with MetS than in those without MetS, with such damage being related to increased levels of inflammation and fibrosis^1^. MetS is independently associated with increased salt sensitivity of blood pressure^2^, and excessive salt intake may contribute to the progression of LV remodeling and diastolic dysfunction in metabolic disorders^3^.

The pathological role of subcutaneous adipose tissue (SAT) in individuals with MetS has remained largely unknown, with visceral adipose tissue (VAT) being more strongly associated with metabolic risk factors than is SAT^4^. Heterogeneity within and between fat depots—in particular, with regard to expression dynamics of uncoupling protein 1 (UCP1)—suggests that SAT and VAT should be considered as distinct types of white fat^5^. In addition, the peroxisome proliferator-activated receptor γ (PPARγ) ligand rosiglitazone preferentially upregulates brown fat-related genes such as those for UCP1 and Cidea in SAT as compared with VAT^6^. Given that SAT function may be modified by increased body and VAT mass, determination of the association of SAT with biomarkers of metabolic and cardiac risk may be clinically important for individuals with obesity^7^. Both the quantity and quality of VAT and SAT have previously been shown to contribute to metabolic risk^8^. A recent study found that insulin sensitivity was improved after subcutaneous liposuction, suggesting that SAT may contribute to MetS pathology^9^. Although numerous studies have shown beneficial outcomes of this procedure in obese humans, the metabolic effects of abdominal liposuction remain unclear^10^.

We have established an animal model of MetS, the DahlS.Z-*Lepr*^fa^*/Lepr*^fa^ (DS/obese) rat, by crossing Dahl salt-sensitive (DS) rats with Zucker rats harboring a missense mutation in the leptin receptor gene (*Lepr*)^11^. DS/obese rats thus develop a MetS-like phenotype that includes salt-sensitive hypertension as well as LV hypertrophy, fibrosis, and diastolic dysfunction, and these changes are associated with increased cardiac oxidative stress and inflammation^12^. Dietary salt restriction attenuates the development of hypertension and LV injury as well as ameliorates inflammation in VAT of these animals^13^. Given that DS/obese rats develop adipocyte hypertrophy and inflammation to similar extents in SAT and VAT^14^, not only VAT but also SAT may play a role in the pathophysiology of MetS and its cardiovascular and other complications in the absence or presence of excess salt. We have now tested this hypothesis by investigating the effects of salt loading and SAT removal on cardiac, renal, and adipose tissue pathology in DS/obese rats.

## Materials and methods

### Animals and experimental protocols

All animal experiments were conducted with the approval of the Animal Experiment Committee of Nagoya University Graduate School of Medicine (Daiko district, approval Nos. 029-028, 030-010, 031-012, and 20014). Seven-week-old male inbred DS/obese rats were obtained from Japan SLC (Hamamatsu, Japan) and were handled in accordance with the guidelines of Nagoya University Graduate School of Medicine as well as with the Guide for the Care and Use of Laboratory Animals (NIH publication No. 85-23, revised 2011). They were allowed free access to normal laboratory chow (CE-2; CLEA Japan, Tokyo, Japan) and drinking water throughout the experimental period. DS/obese rats were randomly assigned to four groups at 8 weeks of age. DS/obese rats subjected to surgical removal of SAT or to sham surgery at 8 weeks of age were provided with 0.3% NaCl as drinking water (MetS + SAT + HS group, *n* = 12 and MetS + HS, *n* = 12 group, respectively) or tap water alone (MetS + SAT group, *n* = 9 and MetS group, *n* = 8, respectively) for 4 weeks from 9 weeks of age. Body weight as well as food and water intake were measured weekly. Systolic blood pressure (SBP) and heart rate (HR) were also monitored as previously described^15^. At 13 weeks of age, rats were placed in metabolic cages for the collection of 24-h urine specimens, and they were subjected to an oral glucose tolerance test (OGTT) and an insulin tolerance test (ITT)^16^. Urine and serum analyses were conducted as described previously^14,17^.

### SAT removal

Inguinal SAT on both sides was removed through a 10- to 20-mm incision in the inguinal region, which was subsequently closed by suturing, in rats anesthetized with isoflurane. As a control, sham surgery was performed with the same incision and suturing but without SAT removal.

### Echocardiography and cardiac catheterization

At 13 weeks of age, rats were anesthetized by intraperitoneal injection of ketamine (50 mg/kg) and xylazine (10 mg/kg) and were subjected to transthoracic echocardiography and cardiac catheterization, as described previously^18^.

### Histology and immunohistochemistry

LV, kidney, VAT, or SAT tissue was analyzed by hematoxylin-eosin (H&E), Azan-Mallory, or periodic acid–Schiff (PAS) staining as well as by immunohistochemical staining for the monocyte–macrophage marker CD68 (antibody clone ED-1, diluted 1:100; Chemicon, Temecula, CA, USA), as previously described^19,20^. The glomerulosclerosis index (GSI) was measured for 50 glomeruli in PAS-stained sections of each rat, also as described previously^21^. The tubulointerstitial injury score (TIS) was evaluated in 10 fields of Azan-Mallory-stained sections for each rat^21^. CD68-positive cells were counted in 20 glomeruli for each rat as previously described^22^. Image analysis was performed with NIH Scion Image software (Scion Corp., Frederick, MD) in a blinded manner to the experimental status of the animals^13^.

### Quantitative RT-PCR analysis

Total RNA was extracted from LV, kidney, or adipose tissue and was subjected to reverse transcription (RT) followed by real-time polymerase chain reaction (PCR) analysis^23^ with primers specific for cDNAs encoding atrial natriuretic peptide (ANP)^24^, monocyte chemoattractant protein-1 (MCP-1)^25^, osteopontin^25^, collagen types I^26^, III^26^, or IV (5′-ATTCCTTTGTGATGCACACCAG-3′ and 5′-AAGCTGTAAGCATTCGCGTAGTA-3′ as forward and reverse primers, respectively; GenBank accession No. NM_00135009.1), connective tissue growth factor (CTGF)^25^, transforming growth factor-β1 (TGF-β1)^24^, adiponectin^27^, angiotensin-converting enzyme (ACE)^24^, the angiotensin II type 1A receptor (AT_1A_R)^24^, the mineralocorticoid receptor (MR)^24^, or serum- and glucocorticoid-regulated kinase 1 (Sgk1)^12^. The expression level of each gene was normalized by that of the glyceraldehyde-3-phosphate dehydrogenase (GAPDH) gene as an internal standard.

### Statistical analysis

Data are presented as means ± SEM. Differences among groups of rats at 13 weeks of age were assessed by one-way factorial analysis of variance (ANOVA) followed by Fisher’s multiple-comparison test. The time courses of body weight, SBP, HR, or food or water intake were compared among groups by two-way repeated-measures ANOVA. Two-way factorial ANOVA was applied to evaluate interaction between salt loading and SAT removal. A *P* value of <0.05 was considered statistically significant.

## Results

### Physiological analysis

**Fig. 1:**
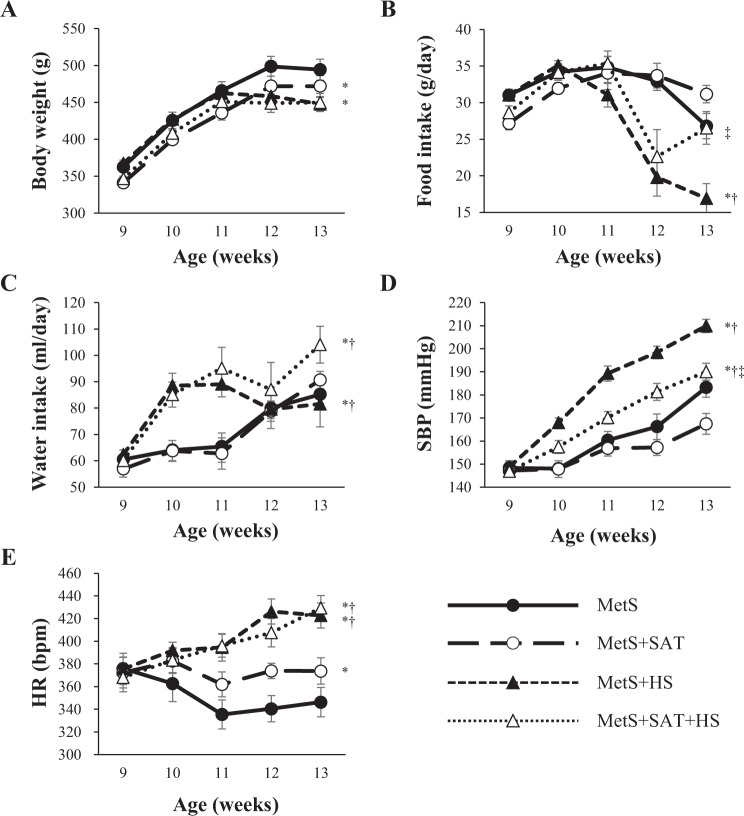
Time courses of body weight, food and water intake, SBP, and HR for rats of the four experimental groups. **a**–**e** Changes in body weight, food intake, water intake, SBP, and HR, respectively. All data are means ± SEM (*n* = 8, 9, 12, and 12 rats in **a**–**e** for MetS, MetS + SAT, MetS + HS, and MetS + SAT + HS groups, respectively). *P < 0.05 vs. MetS, ^†^*P* < 0.05 vs. MetS + SAT, ^‡^*P* < 0.05 vs. MetS + HS.

SAT removal attenuated the increase in body weight in control but not salt-loaded DS/obese rats (Fig. 1a). At 13 weeks of age, body weight was lower in the MetS + HS group than in the MetS group (Table 1). Food intake was lower in the MetS + HS group than in the MetS + SAT + HS group (Fig. 1b). Water intake was initially higher in the MetS + HS group than in the MetS group but was not affected by SAT removal (Fig. 1c). At 13 weeks of age, water intake was lower in the MetS + HS group than in the MetS + SAT + HS group (Table 1). SBP did not differ between the MetS and MetS + SAT groups at 9 weeks of age and thereafter, but it was enhanced by salt loading in both groups (Fig. 1d). This enhancement of SBP apparent in the MetS + HS group was alleviated by SAT removal. HR was also elevated in the MetS + HS group compared with the MetS group, but this elevation was not prevented by SAT removal (Fig. 1e). At 13 weeks of age, the ratios of heart or LV weight to tibial length (indices of cardiac and LV hypertrophy, respectively) were increased in the MetS + HS group compared with the MetS group, but SAT removal had no effect on these parameters (Table 1). Neither salt loading nor SAT removal influenced the ratio of kidney weight to tibial length (Table 1). The ratios of visceral (retroperitoneal or epididymal) fat weight to tibial length were reduced in the MetS + HS group compared with the MetS group, and these effects were prevented by SAT removal (Table 1). Salt loading also reduced subcutaneous (inguinal) fat weight.

**Table 1:** Physiological and biochemical parameters for rats of the four experimental groups at 13 weeks of age.

Parameter	MetS	MetS + SAT	MetS + HS	MetS + SAT + HS
Body weight (g)	494.4 ± 14.3	472.1 ± 14.3	447.8 ± 8.8*	449.9 ± 12.2*
SBP (mmHg)	183.3 ± 4.3	167.4 ± 4.6*	209.9 ± 2.9^*, **^	190.0 ± 3.2^**, ***^
HR (bpm)	346.3 ± 13.0	373.7 ± 11.6	422.8 ± 11.0^*, **^	429.4 ± 11.0^*, **^
Food intake (g/day)	26.8 ± 1.7	31.2 ± 1.2	16.9 ± 2.0^*, **^	26.5 ± 2.2^***^
Water intake (ml/day)	85.1 ± 5.1	90.6 ± 3.3	81.5 ± 8.7	104.0 ± 7.0^***^
Tibial length (TL, mm)	34.0 ± 0.1	33.8 ± 0.2	34.1 ± 0.2	33.7 ± 0.1
Heart weight/TL (mg/mm)	43.8 ± 1.1	41.9 ± 1.7	46.8 ± 0.8^*, **^	48.3 ± 1.0^*, **^
LV weight/TL (mg/mm)	32.8 ± 0.9	31.4 ± 1.3	36.1 ± 0.7^*, **^	37.3 ± 0.9^*, **^
Kidney weight/TL (mg/mm)	112.9 ± 3.9	111.8 ± 4.5	106.3 ± 2.1	113.7 ± 3.3
Epididymal fat weight/TL (mg/mm)	337.7 ± 19.8	355.1 ± 8.9	291.1 ± 7.5^*, **^	338.6 ± 11.4^***^
Retroperitoneal fat weight/TL (mg/mm)	444.3 ± 16.6	439.1 ± 9.4	376.6 ± 16.0^*, **^	451.7 ± 14.4^***^
Inguinal fat weight/TL (mg/mm)	1413.9 ± 57.8		1181.4 ± 77.3*	
OGTT AUC (mg ml^−1^ min)	15782.1 ± 644.0	16095 ± 459.1	17464.6 ± 421.2*	15685.3 ± 586.4^***^
ITT AUC (mg ml^−1^ min)	8560.3 ± 123.4	8262.5 ± 251.6	9928.1 ± 398.3^*, **^	7784.3 ± 334.6^***^
Creatinine clearance (ml/min)	1.71 ± 0.13	1.12 ± 0.18	1.06 ± 0.11*	1.90 ± 0.26^**, ***^
Urinary norepinephrine (µg/day)	0.63 ± 0.08	0.74 ± 0.13	0.97 ± 0.13*	0.97 ± 0.16
Serum IL-6 (pg/ml)	13.5 ± 1.0	9.5 ± 3.6	23.9 ± 3.0^*, **^	12.6 ± 4.5^***^

Data are means ± SEM (*n* = 8, 9, 11, and 12 rats for physiological data and *n* = 5, 5, 6, and 6 rats for biochemical data of MetS, MetS + SAT, MetS + HS, and MetS + SAT + HS groups, respectively).

^*^P < 0.05 vs. MetS.

^**^P < 0.05 vs. MetS + SAT.

^***^P < 0.05 vs. MetS + HS.

### Cardiac function

We performed echocardiography and cardiac catheterization to assess the effects of salt loading and SAT removal on cardiac morphology and function in DS/obese rats. Echocardiography revealed that LV mass, relative wall thickness (RWT), and the thickness of both the interventricular septum (IVST) and LV posterior wall (LVPWT) were increased and that the LV end-diastolic dimension (LVDd) was decreased in the MetS + HS group compared with the MetS group, whereas these changes were not influenced by SAT removal (Table 2). The deceleration time (DcT) and isovolumic relaxation time (IRT), both of which are indices of LV relaxation, were prolonged in the MetS + HS group compared with the MetS group in a manner sensitive to SAT removal (Table 2). Cardiac catheterization showed that LV end-diastolic pressure (LVEDP) was increased in the MetS + HS group compared with the MetS group, and this increase was abolished in the MetS + SAT + HS group (Table 2). The ratio of LVEDP to LVDd, an index of LV diastolic stiffness, showed a pattern of changes similar to that of those for LVEDP.

**Table 2:** Cardiac morphological and functional parameters for rats of the four experimental groups at 13 weeks of age.

Parameter	MetS	MetS + SAT	MetS + HS	MetS + SAT + HS
IVST (mm)	2.46 ± 0.12	2.45 ± 0.08	2.82 ± 0.07^*,**^	2.86 ± 0.04^*,**^
LVPWT (mm)	2.09 ± 0.10	2.08 ± 0.09	2.61 ± 0.07^*, **^	2.58 ± 0.02^*, **^
LVDd (mm)	7.49 ± 0.38	7.10 ± 0.19	6.77 ± 0.19^*^	6.68 ± 0.16^*^
LVDs (mm)	4.27 ± 0.34	3.77 ± 0.10	3.83 ± 0.13	3.66 ± 0.16^*^
LV mass (mg)	1100.4 ± 57.8	1016.9 ± 58.5	1255.3 ± 55.6^*, **^	1236.9 ± 39.7^**^
RWT	0.62 ± 0.06	0.64 ± 0.03	0.81 ± 0.03^*, **^	0.82 ± 0.02^*, **^
LVFS (%)	43.3 ± 2.4	46.8 ± 0.8	43.3 ± 1.4	45.4 ± 1.4
LVEF (%)	81.1 ± 2.4	84.8 ± 0.7	81.5 ± 1.3	83.3 ± 1.2
DcT (ms)	54.7 ± 0.4	49.7 ± 0.8^*^	59.0 ± 1.3^*, **^	53.3 ± 0.8^**, ***^
IRT (ms)	41.4 ± 1.6	40.3 ± 1.4	46.6 ± 1.8^*, **^	42.0 ± 1.9^**^
LVEDP (mmHg)	9.14 ± 1.42	7.83 ± 1.94	14.54 ± 2.25^*, **^	9.82 ± 1.57^***^
LVEDP/LVDd (mmHg/mm)	1.23 ± 0.15	1.13 ± 0.24	2.16 ± 0.54^*, **^	1.42 ± 0.21^***^

Data are means ± SEM (*n* = 7, 9, 10, and 12 rats for MetS, MetS + SAT, MetS + HS, and MetS + SAT + HS groups, respectively). *LVDs* LV end-systolic dimension, *LVFS* LV fractional shortening, *LVEF* LV ejection fraction.

^*^P < 0.05 vs. MetS.

^**^P < 0.05 vs. MetS + SAT.

^***^P < 0.05 vs. MetS + HS.

### Biochemical data

We also examined the effects of salt loading and SAT removal on blood and urine parameters. Glucose tolerance (Table 1) and insulin sensitivity (Table 1) were impaired in the MetS + HS group compared with the MetS group, and the impairment of both parameters was ameliorated by SAT removal. Creatinine clearance measured over 24 h was reduced in the MetS + HS group, but not in the MetS + SAT + HS group, relative to the MetS group (Table 1). Urinary norepinephrine excretion was enhanced by salt loading, but this effect was not inhibited by SAT removal (Table 1). Salt loading increased the serum level of interleukin-6 (IL-6) in a manner sensitive to SAT removal (Table 1).

### Myocardial pathology and gene expression

**Fig. 2:**
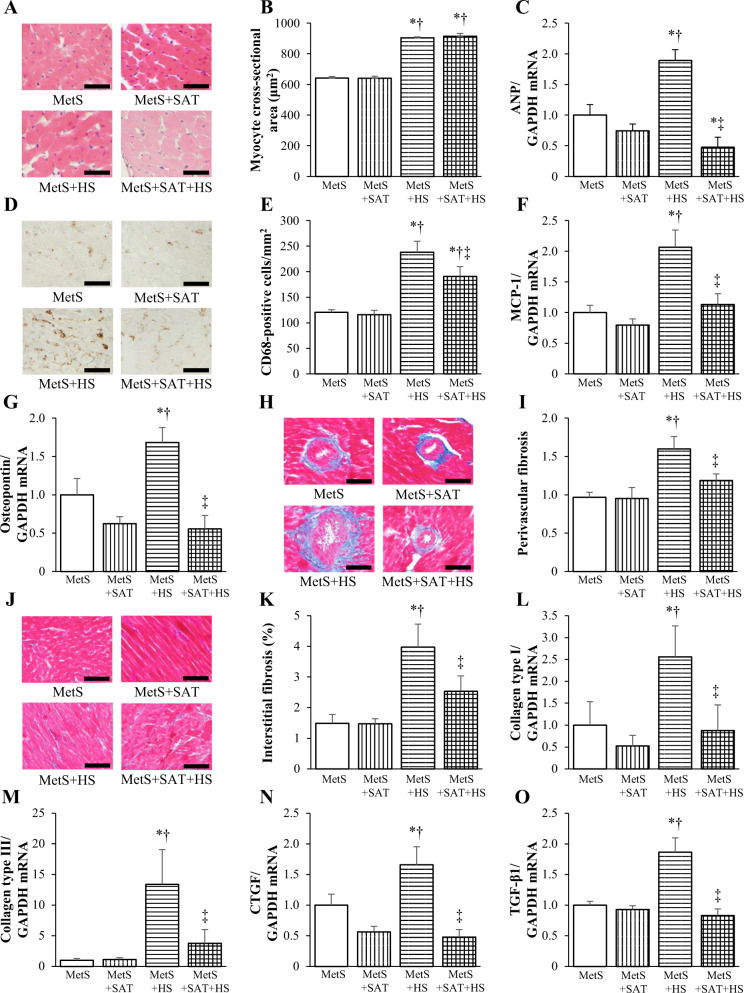
Cardiomyocyte hypertrophy, fibrosis, and inflammation in the left ventricle of rats in the four experimental groups at 13 weeks of age. **a** H&E staining of transverse sections of the LV myocardium (scale bars, 50 μm). **b** Cross-sectional area of cardiac myocytes as determined from sections similar to those in (**a**). **c** Quantitative RT-PCR analysis of relative ANP mRNA abundance. **d** Immunohistochemical analysis of the monocyte–macrophage marker CD68 in transverse sections of the left ventricle (scale bars, 50 μm). **e** Density of CD68-positive cells as determined from sections similar to those in (**d**). **f**, **g** Quantitative RT-PCR analysis of relative MCP-1 and osteopontin mRNA abundance, respectively. **h**, **j** Collagen deposition as revealed by Azan-Mallory staining in perivascular and interstitial regions of the LV myocardium, respectively (scale bars, 100 μm). **i**, **k** Relative extents of perivascular and interstitial fibrosis, respectively, as determined from sections similar to those in (**h**, **j**). **l**‒**o** Quantitative RT-PCR analysis of relative collagen type I or III, CTGF, and TGF-β1 mRNA abundance, respectively. All quantitative data are means ± SEM (*n* = 6 rats for each experimental group). *P < 0.05 vs. MetS, **P < 0.05 vs. MetS + SAT, ***P < 0.05 vs. MetS + HS.

We examined the effects of salt loading and SAT removal on LV injury. LV myocyte cross-sectional area measured in sections stained with H&E was increased by salt loading but was not affected by SAT removal (Fig. 2a, b). Quantitative RT-PCR analysis showed that the amount of ANP mRNA in the left ventricle was higher in the MetS + HS group than in the MetS group, and that this effect of salt loading was prevented by SAT removal (Fig. 2c). Immunostaining for the monocyte–macrophage marker CD68 revealed that the extent of macrophage infiltration in the LV myocardium was increased in the MetS + HS group compared with the MetS group, and that this effect was attenuated by SAT removal (Fig. 2d, e). Expression of the inflammation-related genes for MCP-1 and osteopontin was also upregulated by salt loading in a manner sensitive to SAT removal (Fig. 2f, g). Azan-Mallory staining revealed that fibrosis in perivascular and interstitial regions of the LV myocardium was increased in the MetS + HS group compared with the MetS group, and that this effect was attenuated by SAT removal (Fig. 2h‒k). In addition, expression of the fibrosis-related genes for collagen types I and III, CTGF, and TGF-β1 was upregulated by salt loading, and these effects were suppressed by SAT removal (Fig. 2l‒o).

### Adipose tissue pathology and gene expression

**Fig. 3:**
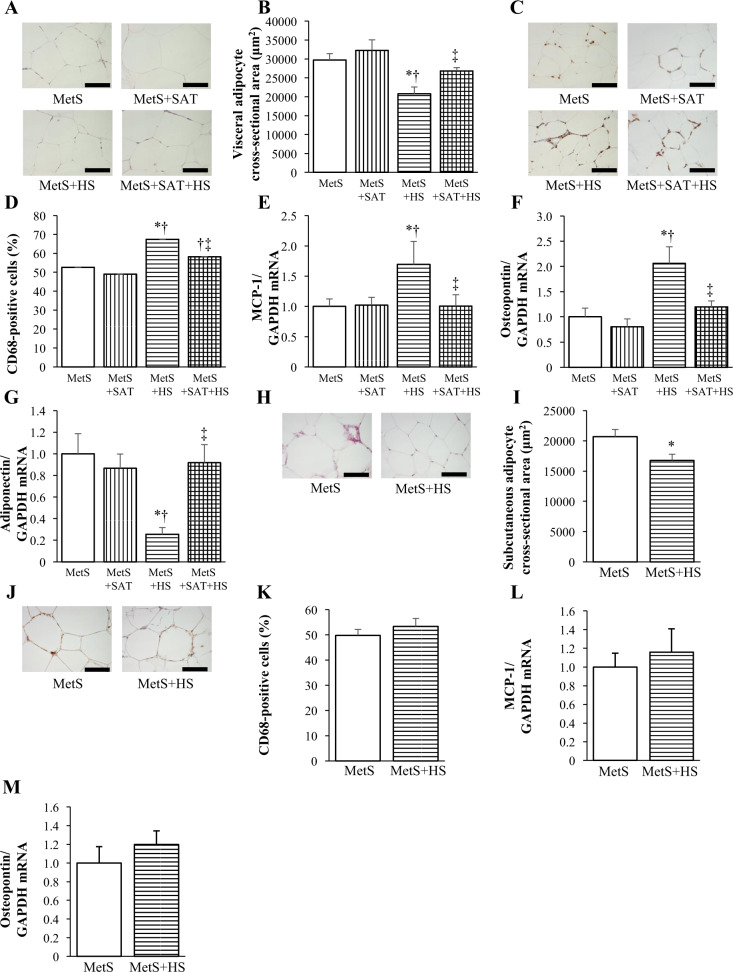
Adipocyte size, macrophage infiltration, and inflammation-related gene expression in VAT and SAT of rats at 13 weeks of age. **a** H&E staining of transverse sections of epididymal adipose tissue (scale bars, 100 μm). **b** Cross-sectional area of epididymal adipocytes as determined from sections similar to those in (**a**). **c** Immunohistochemical analysis of the monocyte–macrophage marker CD68 in transverse sections of epididymal adipose tissue (scale bars, 50 μm). **d** Number of nuclei for CD68-positive cells as a percentage of total nuclei and as determined from sections similar to those in (**c**). **e**‒**g** Quantitative RT-PCR analysis of relative MCP-1, osteopontin, and adiponectin mRNA abundance, respectively. **h** H&E staining of transverse sections of inguinal adipose tissue (scale bars, 100 μm). **i** Cross-sectional area of inguinal adipocytes as determined from sections similar to those in (**h**). **j** Immunohistochemical analysis of CD68 in transverse sections of inguinal adipose tissue (scale bars, 100 μm). **k** Number of nuclei for CD68-positive cells as a percentage of total nuclei and as determined from sections similar to those in (**j**). **l**, **m** Quantitative RT-PCR analysis of relative MCP-1 and osteopontin mRNA abundance, respectively. All quantitative data are means ± SEM (*n* = 6 rats for each experimental group). *P < 0.05 vs. MetS, **P < 0.05 vs. MetS + SAT, ***P < 0.05 vs. MetS + HS.

The cross-sectional area of epididymal (visceral) adipocytes was reduced by salt loading in a manner sensitive to SAT removal (Fig. 3a, b). The percentage of CD68-positive cells in epididymal adipose tissue was increased in the MetS + HS group compared with the MetS group, whereas a similar increase was not apparent in the MetS + SAT + HS group (Fig. 3c, d). Furthermore, the amounts of MCP-1 and osteopontin mRNAs in epididymal fat were upregulated by salt loading in a manner sensitive to SAT removal (Fig. 3e, f). Expression of the adiponectin gene in epididymal fat was reduced in the MetS + HS group relative to the MetS group, and this effect of salt loading was counteracted by SAT removal (Fig. 3g). With regard to subcutaneous fat, salt loading reduced the cross-sectional area of inguinal adipocytes (Fig. 3h, i), similar to its effect on epididymal adipocytes. In contrast to visceral adipose tissue, however, salt loading did not affect the extent of macrophage infiltration (Fig. 3j, k) or the abundance of MCP-1 and osteopontin mRNAs (Fig. 3l, m) in inguinal fat.

### Renal pathology and gene expression

**Fig. 4:**
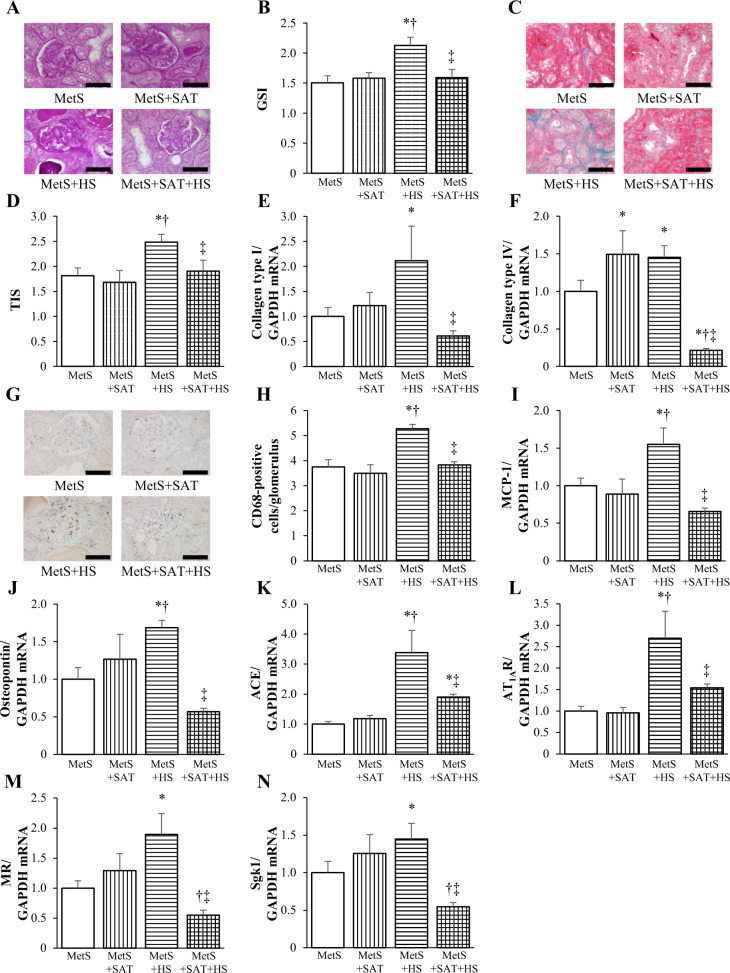
Histological changes, inflammation, and fibrosis- and RAAS-related gene expression in the kidney of rats in the four experimental groups at 13 weeks of age. **a** PAS staining of transverse sections of glomeruli (scale bars, 100 μm). **b** The GSI (range of 0–4 reflects the extent of glomerulosclerosis) as determined from sections similar to those in (**a**). **c** Azan-Mallory staining of transverse sections of tubulointerstitial regions (scale bars, 100 μm). **d** The TIS (range of 0–4 reflects the extent of tubulointerstitial injury) as determined from sections similar to those in (**c**). **e**, **f** Quantitative RT-PCR analysis of relative collagen type I or IV mRNA abundance, respectively. **g** Immunohistochemical analysis of the monocyte-macrophage marker CD68 in transverse sections of glomeruli (scale bars, 100 μm). **h** Number of CD68-positive cells in each glomerulus as determined from sections similar to those in (**g**). **i**‒**n** Quantitative RT-PCR analysis of relative MCP-1, osteopontin, ACE, AT_1A_R, MR, and Sgk1 mRNA abundance, respectively. All quantitative data are means ± SEM (*n* = 6 rats for each experimental group). *P < 0.05 vs. MetS, **P < 0.05 vs. MetS + SAT, ***P < 0.05 vs. MetS + HS.

We also examined the effects of salt loading and SAT removal on renal injury. The GSI, which indicates the extent of glomerulosclerosis (Fig. 4a, b), as well as the TIS, an indicator of the extent of tubulointerstitial injury (Fig. 4c, d), were both higher in the MetS + HS group than in the MetS group, and these effects of salt loading were abolished by SAT removal. Furthermore, the amounts of collagen types I and IV mRNAs were higher in the MetS + HS group than in the MetS group, and these increases were not apparent in the MetS + SAT + HS group (Fig. 4e, f). The number of CD68-positive cells in glomeruli (Fig. 4g, h) as well as expression of the MCP-1 and osteopontin genes in the kidney (Fig. 4i, j) were increased in the MetS + HS group compared with the MetS group, with these increases again being attenuated in the MetS + SAT + HS group. In addition, the amounts of mRNAs for ACE, AT_1A_R, MR, and Sgk1, all of which are related to the renin–angiotensin–aldosterone system (RAAS), were upregulated by salt loading in a manner sensitive to SAT removal (Fig. 4k‒n).

### Interaction between SAT removal and salt loading

All the data generated in the present study were also evaluated by two-way factorial ANOVA to assess the effects of SAT removal and salt loading as well as the possible interaction between these factors (Supplementary Table 1). There was no interaction between SAT removal and salt loading with regard to effects on body weight, SBP, HR, food or water intake, organ weight, or the cross-sectional area of myocytes or visceral adipocytes. A significant interaction was evident for creatinine clearance and insulin tolerance, but not for urinary norepinephrine excretion, serum IL-6 level, or glucose tolerance. With regard to echocardiographic and hemodynamic analyses, a significant interaction was not detected for any of the data shown in Table 2. With respect to histological and gene expression data for the left ventricle, a significant interaction was apparent only for the abundance of ANP, collagen type III, and TGF-β1 mRNAs. Regarding VAT, a significant interaction was detected for adiponectin gene expression. Finally, for the kidney, a significant interaction was apparent for all histological and gene expression parameters with the exception of TIS. The results of two-way factorial ANOVA were thus largely consistent with those obtained by one-way factorial ANOVA presented in Tables 1 and 2 and in Figs. 1–4.

## Discussion

We have shown that, whereas salt loading reduced both SAT and VAT mass in DS/obese rats, it aggravated inflammation in VAT but not in SAT. SAT removal did not prevent sympathetic hyperactivity or LV hypertrophy, but it attenuated the exacerbation of insulin resistance, glucose intolerance, LV injury, and renal injury in salt-loaded DS/obese rats. In contrast, SAT removal in control DS/obese rats did not affect systemic sympathetic activity, dysregulation of glucose metabolism, or LV or renal pathology. Of note, SAT removal also restored fat mass as well as alleviated inflammation and the downregulation of adiponectin gene expression in VAT of salt-loaded DS/obese rats. As far as we are aware, our study is the first to show a pathophysiological role of SAT in salt-exacerbated VAT, cardiac, and renal pathology in MetS.

Although adipose tissue was originally recognized as an energy reservoir to be tapped during periods of starvation, it has subsequently been found to have various endocrine functions. Adipocytes thus secrete adiponectin, which improves insulin sensitivity, whereas hypertrophied adipocytes secrete less adiponectin and more proinflammatory cytokines including MCP-1 and tumor necrosis factor–α (TNF-α). Lipolysis in fat cells is directly or indirectly regulated by many factors including the sympathetic nervous system, nutritional status, and hormones such as catecholamines and insulin^28,29^. Salt intake increases sympathetic nerve activity^30^ and inhibits fat accumulation^31^. We found that urinary norepinephrine excretion, an index of sympathetic activity, was increased by salt loading in DS/obese rats. Moreover, salt loading reduced fat mass and adipocyte size in both SAT and VAT, suggesting that salt-induced sympathetic hyperactivity promoted lipolysis in both these types of adipose tissue. SAT removal did not affect the sympathetic hyperactivity but attenuated the reduction in fat mass and adipocyte size in VAT of salt-loaded DS/obese rats. Two possible reasons for the effects of SAT removal on VAT morphology are that food intake was higher in the MetS + SAT + HS group than in the MetS + HS group, or that excess energy was stored in VAT instead of SAT.

MCP-1, which plays a key role in macrophage infiltration, is released in increased amounts from hypertrophied adipocytes as a result of dysregulation of adipocytokine secretion. Macrophages manifest M1 or M2 phenotypes, which are proinflammatory and anti-inflammatory, respectively. M1 macrophages and adipocytes functionally interact with each other. Free fatty acids (FFAs) generated by lipolysis and released from adipocytes thus promote the secretion of TNF-α from M1 macrophages, and TNF-α then acts on adipocytes to increase the release of FFAs as well as of MCP-1, TNF-α, and IL-6, with this vicious circle giving rise to adipose tissue inflammation^32^. Mouse and human macrophages exposed to high salt secrete more proinflammatory and less anti-inflammatory cytokines than do those exposed to normal salt concentrations^33^. In the present study, macrophage infiltration in VAT was increased in the MetS + HS group compared with the MetS group and this increase was accompanied by upregulation of MCP-1 and osteopontin gene expression. SAT removal attenuated these changes, indicating that salt loading promoted VAT inflammation in a manner sensitive to SAT removal. In addition, salt loading downregulated adiponectin gene expression in VAT as well as increased the IL-6 concentration in serum, with both of these effects also being attenuated by SAT removal. Despite these beneficial effects of SAT removal, salt loading did not increase inflammation in SAT, in contrast to its proinflammatory effects in VAT. It is possible that the reduction in VAT mass due to salt-induced lipolysis was counteracted by compensatory fat accumulation in VAT after SAT removal, resulting in reduced production and release of proinflammatory cytokines in the MetS + SAT + HS group in comparison with the MetS + HS group.

Insulin signaling activity in adipocytes is inhibited by inflammation^34,35^. High concentrations of FFAs in plasma also promote insulin resistance, possibly as a result of an FFA-induced increase in circulating MCP-1 levels and macrophage infiltration into adipose tissue^36–38^. The abundance of adiponectin mRNA in adipocytes and the plasma adiponectin concentration are both reduced by TNF-α^39^, which might be expected to impair insulin sensitivity^40^. Our ITT data suggest that insulin resistance was exacerbated by salt loading and that SAT removal prevented this exacerbation in DS/obese rats, consistent with the notion that VAT pathology plays a pivotal role in the development of insulin resistance. Our OGTT data also showed that glucose intolerance was worsened in association with salt-induced exacerbation of insulin resistance. These observations suggest that the changes in insulin sensitivity and glucose tolerance induced by salt loading and SAT removal may be attributable to those in the expression of genes for proinflammatory cytokines and adiponectin in VAT.

Dietary salt is thought to be a major cause of increased blood pressure^41^. Insulin resistance and consequent hyperinsulinemia^40^ as well as proinflammatory cytokines^42^ also play important roles in obesity-related hypertension. Whereas obesity and essential hypertension are both characterized by sympathetic overactivity, certain features of such altered activity in human obesity and obesity-related hypertension differ from those in essential hypertension^43^. Most notably, elevated sympathetic activity in obesity often occurs in the absence of hypertension, suggestive of regional differences in sympathetic nerve activity among some pathological states. The lack of a difference in urinary norepinephrine excretion between the MetS + HS and MetS + SAT + HS groups thus does not necessarily indicate that cardiac sympathetic activity was not altered by SAT removal in salt-loaded DS/obese rats. Although LV (and cardiomyocyte) hypertrophy is primarily load dependent^44,45^, it is also influenced by sympathetic overactivity and other obesity-related factors^46–48^. We observed that SBP was higher in the MetS + HS group than in the MetS group, and that indices of both LV (and cardiomyocyte) hypertrophy and concentricity were increased by salt loading. In spite of the alleviation of salt-exacerbated hypertension by SAT removal, the extent of LV hypertrophy did not differ between the MetS + HS and MetS + SAT + HS groups, suggesting that LV hypertrophy and concentricity were influenced not only by load reduction but also by other factors such as a possible increase in cardiac sympathetic activity, as would be expected to occur in response to the observed increase in water (0.3% NaCl) intake in the MetS + SAT + HS group.

High blood pressure contributes to myocardial injury^49^. Cardiac inflammation is also a potential cause of cardiac fibrosis and consequent LV diastolic dysfunction^50^. We have previously shown that DS/obese rats develop LV inflammation and fibrosis as well as diastolic dysfunction, and that these changes are alleviated by dietary salt restriction^13^. In the present study, we found that salt loading exacerbated LV inflammation, fibrosis, and diastolic dysfunction as well as systemic inflammation (as revealed by the serum concentration of IL-6), and that all of these effects were suppressed by SAT removal. The reduction in the circulating IL-6 level induced by SAT removal in salt-loaded DS/obese rats thus occurred in parallel with alleviation of inflammation in VAT. These data suggest that salt-exacerbated LV injury in this model of MetS is likely attributable in part to blood pressure elevation as well as to systemic inflammation associated with increased production of proinflammatory cytokines in VAT. ANP has a variety of cardioprotective effects^51^, and its expression in the heart closely correlates with LVEDP^52^. The observed inhibition of the salt-induced upregulation of ANP gene expression in the heart by SAT removal was probably due to the amelioration of LV injury despite the unchanged presence of LV hypertrophy.

The RAAS has been associated with hypertension, obesity, and MetS. Angiotensin II induces the contraction of systemic arteries and the release of aldosterone from the adrenal cortex. Aldosterone promotes sodium retention in the body, which results in an increase in the circulating blood volume and consequent increased cardiac output and peripheral vascular resistance. A high-salt diet in DS rats was found to promote hypertension and to reduce plasma renin activity and the circulating angiotensin II concentration without affecting angiotensin II abundance in the kidney^53^. We have now found that expression of RAAS-related genes in the kidney was increased in the MetS + HS group compared with the MetS group, consistent with the notion that the renal RAAS operates independently of the plasma RAAS. Furthermore, our evaluation of renal morphology, function, and inflammation indicated that renal dysfunction and pathology were worsened by salt loading and that these effects were prevented by SAT removal, suggesting that pressure overload or systemic inflammation influenced renal pathology and function. Renal sympathetic activity has recently been recognized as a potential therapeutic target for the management of resistant hypertension^54^. Although renal denervation reduced blood pressure, it did not affect salt sensitivity, in rats deficient in endothelin receptor type B^55^. In addition, whereas arterial pressure was lowered in Sprague-Dawley rats by renal denervation, it was increased to a similar extent in both denervated and intact animals by increased salt intake^56^. Renal injury may thus be related to blood pressure and systemic inflammation, but renal sympathetic overactivity is unlikely a major determinant of salt-induced exacerbation of hypertension in DS/obese rats. However, we cannot rule out the possibility that renal damage preceded the appearance of hypertension in DS/obese rats. Given that adiponectin mitigates the effects of angiotensin II in the kidney^57^, the inhibition of the salt-induced downregulation of adiponectin gene expression in VAT by SAT removal may have contributed, at least in part, to the associated amelioration of renal injury and dysfunction.

There are a few limitations in this study. First, we have not established the causal relationship between the progression of hypertension and renal damage in salt-loaded DS/obese rats. Kidney dysfunction is thought to play an essential role in the development of salt-sensitive hypertension^58^. Moreover, sodium accumulates to a greater extent in the skin and skeletal muscle than in plasma of animals and humans with hypertension, which can lead to renal and vascular inflammation through activation of immune cells such as macrophages and T cells^59^. Renal sympathetic denervation in DS/obese rats may thus provide insight into the causal relation between hypertension and renal injury, given that urinary norepinephrine excretion does not necessarily correlate with renal sympathetic activity. Second, our data do not provide mechanistic insight into the attenuation of hypertension and renal pathology by SAT removal in salt-loaded DS/obese rats. The renal/adipose–brain–peripheral sympathetic reflex might have contributed to the salt-induced exacerbation of cardiac, renal, and visceral fat pathology in such rats^60^. Future studies are required to clarify the detailed molecular mechanisms for this effect.

We have here shown that salt-induced inflammatory responses in VAT and LV tissue as well as in the systemic circulation were accompanied by deterioration of insulin resistance, glucose intolerance, as well as LV fibrosis and diastolic dysfunction. Given that all of these effects of salt loading were suppressed by SAT removal, not only VAT but also SAT may play a role in the salt-induced exacerbation of MetS pathophysiology and associated organ damage. The observed attenuation of hypertension and renal pathology by SAT removal in salt-loaded DS/obese rats suggests that SAT contributes to the salt sensitivity of blood pressure. In conclusion, SAT is associated with salt-induced exacerbation of MetS and cardiac and renal pathology in DS/obese rats, and the beneficial effects of SAT removal are at least in part due to inhibition of salt-exacerbated VAT and systemic inflammation.

## Supplementary information

Supplementary information
