# Supplementary material for: Alleviation of salt-induced exacerbation of cardiac, renal, and visceral fat pathology in rats with metabolic syndrome by surgical removal of subcutaneous fat
Source: Nutr Diabetes. 2020 Aug 10;10:28. doi: 10.1038/s41387-020-00132-1 (PMC7417575; doi:10.1038/s41387-020-00132-1)
Supplement: Supplementary file 1 — Supplementary information [file 41387_2020_132_MOESM1_ESM.docx]

**Supplementary Table 1 Two-way factorial ANOVA for the effects of salt loading and SAT removal in rats of the four experimental groups at 13 weeks of age**

| **Parameter** | **SAT removal** | | **Salt loading** | | **Interaction** | |
| --- | --- | --- | --- | --- | --- | --- |
|  | ***F* value** | ***P* value** | ***F* value** | ***P* value** | ***F* value** | ***P* value** |
| Body weight (g) | 0.669 | 0.4188 | 7.756 | 0.0084 | 0.973 | 0.3303 |
| SBP (mmHg) | 21.372 | <0.0001 | 40.71 | <0.0001 | 0.275 | 0.6031 |
| HR (bpm) | 2.077 | 0.1587 | 31.378 | <0.0001 | 0.772 | 0.3857 |
| Food intake (g/day) | 12.278 | 0.0012 | 13.248 | 0.0008 | 1.745 | 0.1946 |
| Water intake (ml/day) | 3.913 | 0.0559 | 0.479 | 0.4931 | 1.46 | 0.2348 |
| Tibial length (TL, mm) | 3.946 | 0.0544 | 0.0004 | 0.9834 | 0.484 | 0.4909 |
| Heart weight/TL (mg/mm) | 0.033 | 0.8559 | 18.09 | 0.0001 | 2.418 | 0.1284 |
| LV weight/TL (mg/mm) | 0.007 | 0.9336 | 24.122 | <0.0001 | 1.943 | 0.1716 |
| Kidney weight/TL (mg/mm) | 0.889 | 0.3522 | 0.505 | 0.4822 | 1.617 | 0.2119 |
| Epididymal fat weight/TL (mg/mm) | 8.281 | 0.0076 | 7.827 | 0.0092 | 1.769 | 0.1942 |
| Retroperitoneal fat weight/TL (mg/mm) | 16.112 | 0.0003 | 12.505 | 0.0012 | 0.729 | 0.3993 |
| Creatinine clearance (ml/min) | 0.466 | 0.5026 | 0.134 | 0.7187 | 15.621 | 0.0008 |
| Urinary norepinephrine (µg/day) | 0.019 | 0.8929 | 6.13 | 0.0215 | 0.461 | 0.5041 |
| Serum IL-6 (pg/ml) | 6.056 | 0.0249 | 4.804 | 0.426 | 1.4 | 0.2531 |
| OGTT AUC (mg ml­^–1^ min) | 1.908 | 0.1785 | 1.438 | 0.241 | 3.884 | 0.0591 |
| ITT AUC (mg ml­^–1^ min) | 11.566 | 0.003 | 1.535 | 0.2304 | 6.612 | 0.0187 |
| IVST (mm) | 0.043 | 0.8374 | 29.533 | <0.0001 | 0.153 | 0.6984 |
| LVPWT (mm) | 0.073 | 0.7884 | 67.068 | <0.0001 | 0.034 | 0.8541 |
| LVDd (mm) | 1.272 | 0.2673 | 7.433 | 0.0101 | 0.534 | 0.4699 |
| LVDs (mm) | 3.807 | 0.0593 | 2.614 | 0.1152 | 0.932 | 0.3412 |
| LV mass (mg) | 1.036 | 0.3159 | 14.008 | 0.0007 | 0.422 | 0.5204 |
| RWT | 0.265 | 0.6103 | 32.947 | <0.0001 | 0.022 | 0.884 |
| LVFS (%) | 3.708 | 0.0625 | 0.222 | 0.6402 | 0.287 | 0.5957 |
| LVEF (%) | 4.092 | 0.051 | 0.187 | 0.6682 | 0.481 | 0.4926 |
| DcT (ms) | 36.043 | <0.0001 | 19.904 | <0.0001 | 0.117 | 0.7347 |
| IRT (ms) | 2.699 | 0.1096 | 3.951 | 0.0549 | 1.085 | 0.305 |
| LVEDP (mmHg) | 4.832 | 0.0452 | 6.7 | 0.215 | 2.105 | 0.1688 |
| LVEDP/LVDd (mmHg/mm) | 6.677 | 0.0239 | 11.019 | 0.0061 | 4.634 | 0.0524 |
| **Left ventricle** |  |  |  |  |  |  |
| Myocyte cross-sectional area (μm^2^) | 0.12 | 0.7338 | 426.99 | <0.0001 | 0.154 | 0.7001 |
| ANP/GAPDH mRNA | 34.767 | <0.0001 | 4.768 | 0.0496 | 16.72 | 0.0015 |
| CD68-positive cells/mm^2^ | 2.868 | 0.1097 | 38.993 | <0.0001 | 1.903 | 0.1867 |
| MCP-1/GAPDH mRNA | 9.888 | 0.0038 | 14.96 | 0.0006 | 4.1 | 0.0522 |
| Osteopontin/GAPDH mRNA | 16.72 | 0.0008 | 2.781 | 0.1137 | 4.152 | 0.575 |
| Perivascular fibrosis | 3.091 | 0.0978 | 12.744 | 0.0026 | 2.643 | 0.1236 |
| Interstitial fibrosis (%) | 2.281 | 0.1426 | 13.44 | 0.0011 | 2.174 | 0.1519 |
| Collagen type I/GAPDH mRNA | 4.232 | 0.0575 | 3.336 | 0.0877 | 1.33 | 0.2668 |
| Collagen type III/GAPDH mRNA | 8.815 | 0.009 | 22.25 | 0.0002 | 9.306 | 0.0076 |
| CTGF/GAPDH mRNA | 18.837 | 0.0002 | 2.377 | 0.1352 | 4.008 | 0.0558 |
| TGF-β1/GAPDH mRNA | 20.69 | <0.0001 | 9.864 | 0.0038 | 15.798 | 0.0004 |
| **VAT** |  |  |  |  |  |  |
| Adipocyte cross-sectional area (μm^2^) | 4.921 | 0.0414 | 13.769 | 0.0019 | 0.822 | 0.378 |
| CD68-positive cells (%) | 7.897 | 0.126 | 27.706 | <0.0001 | 1.541 | 0.2324 |
| MCP-1/GAPDH mRNA | 3.255 | 0.0816 | 3.317 | 0.0789 | 3.676 | 0.0651 |
| Osteopontin/GAPDH mRNA | 7.67 | 0.0099 | 14.464 | 0.0007 | 3.017 | 0.0934 |
| Adiponectin/GAPDH mRNA | 3.339 | 0.0764 | 5.759 | 0.022 | 7.62 | 0.0092 |
| **Kidney** |  |  |  |  |  |  |
| GSI | 3.019 | 0.0911 | 5.914 | 0.0203 | 5.507 | 0.0247 |
| TIS | 3.081 | 0.0877 | 4.769 | 0.0356 | 1.199 | 0.2807 |
| Collagen type I/GAPDH mRNA | 4.039 | 0.0524 | 0.619 | 0.437 | 7.215 | 0.0111 |
| Collagen type IV/GAPDH mRNA | 6.334 | 0.0181 | 7.79 | 0.0095 | 34.183 | <0.0001 |
| CD68-positive cells/glomerulus | 14.728 | 0.0005 | 17.427 | 0.0002 | 7.217 | 0.011 |
| MCP-1/GAPDH mRNA | 11.289 | 0.0019 | 1.129 | 0.295 | 6.811 | 0.0131 |
| Osteopontin/GAPDH mRNA | 6.963 | 0.0136 | 0.001 | 0.9773 | 18.447 | 0.0002 |
| ACE/GAPDH mRNA | 4.575 | 0.0397 | 26.333 | <0.0001 | 7.633 | 0.0092 |
| AT_1A_R/GAPDH mRNA | 5.115 | 0.0302 | 18.628 | 0.0001 | 4.436 | 0.0426 |
| MR/GAPDH mRNA | 5.612 | 0.0233 | 0.121 | 0.7303 | 13.592 | 0.0007 |
| Sgk1/GAPDH mRNA | 1.705 | 0.2003 | 0.024 | 0.8773 | 16.531 | 0.0003 |
